# Genome-scale definition of the transcriptional programme associated with compromised PU.1 activity in acute myeloid leukaemia

**DOI:** 10.1038/leu.2015.172

**Published:** 2015-07-21

**Authors:** J I Sive, S Basilico, R Hannah, S J Kinston, F J Calero-Nieto, B Göttgens

**Affiliations:** 1 Department of Haematology, Cambridge Institute for Medical Research and Wellcome Trust and MRC Cambridge Stem Cell Institute, University of Cambridge, Cambridge, UK

## Abstract

Transcriptional dysregulation is associated with haematological malignancy. Although mutations of the key haematopoietic transcription factor *PU.1* are rare in human acute myeloid leukaemia (AML), they are common in murine models of radiation-induced AML, and *PU.1* downregulation and/or dysfunction has been described in human AML patients carrying the fusion oncogenes RUNX1-ETO and PML-RARA. To study the transcriptional programmes associated with compromised PU.1 activity, we adapted a *Pu.1*-mutated murine AML cell line with an inducible wild-type PU.1. PU.1 induction caused transition from leukaemia phenotype to monocytic differentiation. Global binding maps for PU.1, CEBPA and the histone mark H3K27Ac with and without PU.1 induction showed that mutant PU.1 retains DNA-binding ability, but the induction of wild-type protein dramatically increases both the number and the height of PU.1-binding peaks. Correlating chromatin immunoprecipitation (ChIP) Seq with gene expression data, we found that PU.1 recruitment coupled with increased histone acetylation induces gene expression and activates a monocyte/macrophage transcriptional programme. PU.1 induction also caused the reorganisation of a subgroup of CEBPA binding peaks. Finally, we show that the PU.1 target gene set defined in our model allows the stratification of primary human AML samples, shedding light on both known and novel AML subtypes that may be driven by PU.1 dysfunction.

## Introduction

Production of mature haematopoietic cells is regulated by a complex network of transcription factors (TFs), which together with epigenetic regulators drive cellular decision-making processes in response to external cues. Disturbance of these networks can lead to malignant transformation, for example, RUNX1 and its binding partner CBFB control multiple aspects of normal haematopoiesis, but fusion proteins such as RUNX1-ETO or CBFB-MYH11 can cause acute myeloid leukaemia (AML).^1, 2^

PU.1 is an ETS-family TF that has multiple roles in normal haematopoiesis. Structurally, it consists of a transactivating region, a middle PEST region involved in protein–protein interactions particularly with the IRF TFs and a C-terminal DNA-binding domain.^3^ PU.1 was recently shown to contribute to the early establishment of haematopoietic transcriptional programmes,^4^ and is known to have a key role during foetal haematopoiesis.^5, 6^ In the adult, PU.1 functions as a major determinant of both myeloid and B-cell lineages^7, 8^ with its effect often modulated by interactions with other TFs such as CEBPA,^9^ GATA1^10^ and c-JUN.^11^ More recently, PU.1 has been identified as a major regulator of cell cycle both in blood stem cells and during myeloid maturation.^12, 13^

Loss of PU.1 in murine models can cause AML, but the precise relationship and mechanism remains unclear. Germ-line knockouts of *Pu.1* cause lethality either *in utero*^5^ or soon after birth,^6, 14^ whereas partial knockdown to levels of 20% by deletion of the *Pu.1*-14 Upstream Regulatory Element is compatible with foetal life, but causes AML within 3–8 months. This had led to the theory that residual PU.1 activity is required to promote leukaemogenesis.^15^ However, an alternative model with somatic *Pu.1* deletion causing a complete loss of protein also causes AML,^16^ and *Pu.1* hypomorphic mutants are frequently observed in radiation-induced mouse models of AML.^17, 18, 19, 20^ This suggests that any loss of PU.1 below a certain threshold may be sufficient for leukaemogenesis;^21^ however, a recent report suggests that some PU.1 function/levels are needed to maintain stem cell function of leukaemic cells.^22^

Although cases of *PU.1* and Upstream Regulatory Element mutations have been recorded in human AML, they are much rarer than in mice.^23, 24, 25, 26, 27^ Better described are mechanisms suppressing PU.1 function, such as disruption of PU.1 transactivation activity by RUNX1-ETO,^11^ or interference with PU.1 binding (including at its own promoter site) by PML-RARA.^28^ Importantly, there has as yet been no specific investigation into the transcriptional programmes associated with the loss of PU.1 activity in AML.

To investigate the effects of restoring wild-type PU.1 to a PU.1 mutant leukaemia model, we developed an inducible PU.1 system, and showed that the induction of wild-type protein causes transit from the leukaemic state to monocytic differentiation. Using chromatin immunoprecipitation (ChIP)-Seq, we produced genome-scale maps of DNA binding by PU.1, the associated TF CEBPA and the H3K27Ac histone mark before and after PU.1 induction, and complemented these with gene expression profiling data. Unexpectedly, mutant PU.1 was bound to a large number of genomic regions, but induction of wild-type PU.1 resulted in a substantial expansion of binding sites, a subset of which was associated with elevated histone H3K27 acetylation, which in turn correlated with increased expression of nearby target genes. Our results also show that *de novo* binding of wild-type PU.1 has the capacity to recruit CEBPA to a subset of new sites. Finally, we show that the PU.1 target gene set in our model can be utilised to stratify primary human AML samples, shedding light on both known and novel AML subtypes that may be driven by PU.1 dysfunction.

## Materials and Methods

### Cell culture

X18.1.1 cells were maintained in high glucose RPMI 1640 supplemented with 10% foetal calf serum, 1% penicillin/streptomycin, 300 μM asparagine, 2 mM L-glutamine and 50μM beta-mercaptoethanol. Cells were partially adherent requiring trypsin treatment for passage, and were maintained at 3–10 × 10^5^ cells/ml.

### DNA and RNA

Genomic DNA was purified by phenol-chloroform extraction. RNA was extracted using Tri-Reagent (Sigma, Dorset, UK) and then treated with DNase I (Ambion, Paisley, UK), following the manufacturer's specifications. For DNA sequencing, exonic fragments of endogenous Pu.1 were amplified by PCR and cloned into the PGEM-T Easy vector (Promega, Southampton, UK). Both strands of all five exons were sequenced and compared with the Pu.1 sequence of reference (obtained from Ensembl). Integration of PuER was confirmed by PCR using primers encompassing the Pu.1-ERTM fusion region. All primers used are listed in Supplementary Table 1.

### Viral transduction

X18.1.1 cells were transduced with PuER or empty vector (EV) control using retrovirus produced with Psi-Eco Retrovirus packaging vector (Clontech, Saint-Germain-en-Laye, France) in 293 T cells. Cells were infected by centrifugation in the presence of polybrene and then selected with 0.5 μg/ml puromycin. Clonal cell lines were obtained by limiting dilution and further expanded. 4-Hydroxytamoxifen (OHT) (Sigma) inductions were carried out at 100 nM.

### Fluorescence-activated cell sorting and cell proliferation assays

Flow cytometry was performed on a BD LSRFortessa (Oxford, UK) cell analyser using the following antibodies: CD11b (BioLegend, London, UK; 101212) and F4/80 (BioLegend, 123113). Cell proliferation was assayed by counting live cells following Trypan Blue exclusion.

### Immunoblotting

Protein lysate in modified RIPA buffer (50 mM TriS pH7.4, 150 mM NaCl, 1% NP40, 0.25% Na deoxycholate, 1 mM EDTA) was run on a 12% sodium dodecyl suphate polyacrylamide gel, and transferred to polyvinylidene fluoride membrane by overnight wet blotting. Membranes were probed using primary antibodies against ERα (Santa Cruz Biotechnologies, Heidelberg, Germany; sc542x), PU.1 (Santa Cruz Biotechnologies, sc352x) and β-actin (Sigma, A5441).

### ChIP sequencing

ChIP was performed as previously described^29^ using the following antibodies: PU.1 (Santa Cruz Biotechnologies, sc352x), CEBPA (Santa Cruz Biotechnologies, sc61x), H3AcK27 (Abcam, Cambridge, UK; ab4729) and rabbit IgG (Sigma, I5006). Library construction was performed using the Illumina TruSeq DNA Sample Kit (Illumina, Cambridge, UK) according to the manufacturer's instructions. Sequencing was performed on the Illumina HiSeq 2000 platform. Reads were mapped to the mm10 mouse reference genome using Bowtie2.^30^ Mapped reads were converted to density plots and displayed as UCSC genome browser custom tracks, and peaks called using MACS2.^31^

Using BEDTools,^32^ ChIP-Seq peak coordinates were combined between PU.1− and PU.1+ conditions for each TF ChIP, and peaks overlapping by at least 1 bp were merged. Coverage scores were counted using the intersectBed function for each merged peak region, and then normalised for peak length and total read counts (per 1 million reads). For H3K27Ac, read coverage regions were extended to 800 bp, and then normalised as above.

### Microarray gene expression analysis

Triplicate samples were amplified using TotalPrep 96-RNA amplification kit (Applied Biosystems, Paisley, UK/Ambion) and hybridised to MouseWG-6v2 microarrays (Illumina) by Cambridge Genomic Services. Raw data were filtered in R to remove any probes with detection call ⩾0.01 in all samples, and then transformed by variance-stabilising transformation in the *lumi* package.^33^ Data were quantile-normalised, and differentially expressed probes identified using the *limma* package.^34^ Probes with adjusted *P*-value⩽0.05, and fold change>1.2 were considered significant.

### Data analysis

Normalised ChIP-Seq read counts were combined into one matrix and used for subsequent differential score calculations and correlation analyses, as well as integration of ChIP-Seq and gene expression data. All analysis was performed in R with plots created using *ggplot2*.^35^

Peaks were assigned to genes using annotation from UCSC. Peaks within regions contained in the mammalian promoter database MPromdDb were allocated to promoter or within the gene body to intragenic. The remaining intergenic peaks were assigned to both the nearest 5' and 3' genes. If no gene was found within 50 kb of an intergenic peak, it was not assigned to any gene. *De novo* motif analysis on the central 80 bp peak regions was performed using Homer.^36^ Only significant motifs (q-value⩽0.01) with ⩾5% peak coverage were reported. Geneset enrichment analyses were performed using Enrichr.^37^ ChIP-Seq peaks were compared with those of primary macrophages (GSM537983)^36^ using the Codex webtool.^38^ PU.1 target genes were used for unsupervised analysis of published expression arrays (GSE34723 (ref. 39) and GSE6891 (ref. 40)) in R with the *pvclust* package.^41^ Heatmaps were generated using the *heatmap.2* function from the gplots package.^42^

### GEO data deposition

The raw and processed data from the ChIP-Seq and microarray experiments reported in this publication have been submitted to the NCBI Gene Expression Omnibus (www.ncbi.nlm.nih.gov/geo) and assigned the identifier GSE63317.

## Results

### Establishment and validation of an inducible rescue model of leukaemia dependent on loss of functional PU.1

**Figure 1:**
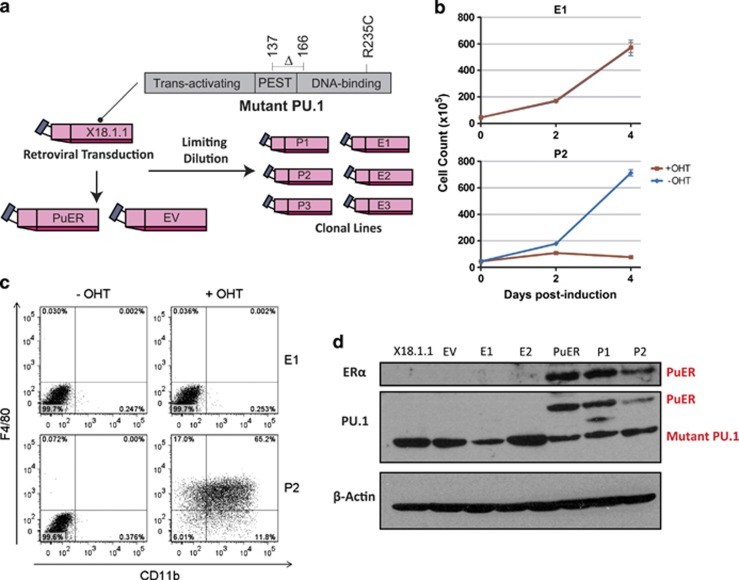
Production and validation of PuER-inducible cell lines. (**a**) Parental cell line X18.1.1 contains a hemizygous deletion of one Pu.1 allele. The remaining allele carries an R235C substitution in the DNA-binding domain and a deletion (137–166) involving the PEST domain. X18.1.1 cell line was retrovirally transduced with MSCV-puro-PuER or control MSCV-puro EV. After puromycin selection, clonal lines were obtained by limiting dilution. (**b**) Cell growth was specifically arrested in PuER line after 4 days of OHT incubation. Cell growth was monitored for two selected clonal lines, EV (E1) and PuER (P2), during 4 days in presence (+ OHT) or absence (−OHT) of OHT. Mean and standard error of the mean (s.e.m.) for two different experiments (each one performed in triplicate) are shown. (**c**) Restoration of wild-type PU.1 induces the upregulation of CD11b and F4/80 surface markers. Fluorescence-activated cell sorting results corresponding to CD11b and F4/80 surface expression in EV (E1) and PuER (P2) clonal lines after 4 days in presence (+ OHT) or absence (−OHT) of OHT are shown. (**d**) PuER protein detection by western blot. Mutant (endogenous) PU.1 protein can be detected in all samples (parental X18.1.1 line, EV and PuER bulk populations and selected clonal lines E1, E2, P1 and P2) using an antibody against PU.1. PUER fusion protein could only be detected in PuER bulk populations and selected clonal lines P1 and P2 when using antibodies against ERα and PU.1. Detection of β-actin was used a control.

To investigate the role of reduced PU.1 in AML, we utilised a radiation-induced murine AML cell line X18.1.1,^18^ characterised by hemizygous deletion of one Pu.1 allele, and two missense mutations in the remaining allele: an R235C substitution within the DNA-binding domain and a 30 amino acid deletion affecting the PEST domain (Figure 1a). We confirmed these mutations by resequencing both genomic and cDNA.

Enforced expression of wild-type PU.1 in X18.1.1 cells has previously been shown to inhibit clonogenic growth, induce monocytic differentiation and elicit apoptosis.^18^ To study the mechanisms underlying this PU.1-induced exit from a leukaemic transcriptional programme, we virally transduced cells with either an OHT-inducible Pu.1-ERTM fusion gene (PuER)^43^ or a control EV within an MSCV-puro retroviral packaging system (Figure 1a).

Incubation in 100 nM OHT caused marked cessation of cell proliferation in liquid culture, reduction of CD117 expression and increased expression of the cell surface markers CD11b and F4/80, consistent with maturation towards a monocytic phenotype (Supplementary Figures 1a and b). We then obtained clonal populations produced by limiting dilution and individual clones with minimal ‘leakage' (PU.1 activity without OHT induction) were selected for further investigation. Clonal populations showed the same characteristics (Figures 1b and c and Supplementary Figure 1c).

The stable integration of PuER was confirmed in PuER lines by PCR, PU.1 mRNA levels quantified by qPCR in PuER lines (Supplementary Figure 2) and PuER protein identified by immunoblotting with anti-ERα antibody (Figure 1d). Anti-PU.1 antibody confirmed the identity of the detected band, and interestingly also detected substantial signal at the size expected for endogenous PU.1 in both control and PuER-transduced cells. This demonstrates that the Pu.1 mutations do not prevent protein translation, and suggests that the phenotype is the result of functional deficiencies in genomic PU.1 binding.

### Induction of wild-type PU.1 causes qualitative and quantitative changes to PU.1 binding

**Figure 2:**
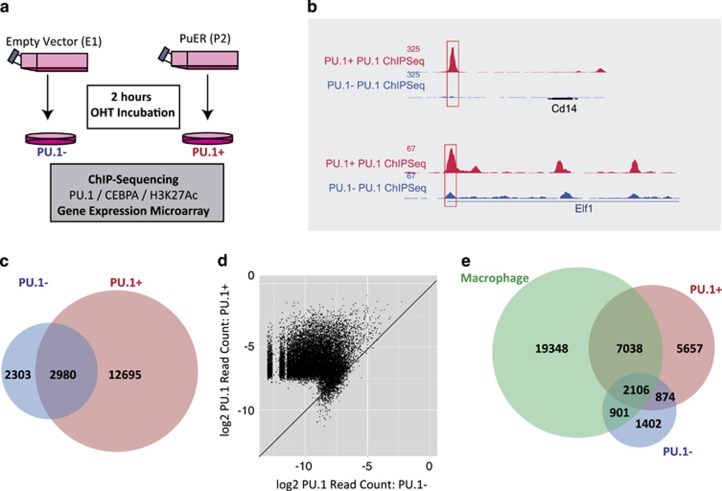
PU.1 ChIP-Seq binding profiles following wild-type PU.1 induction. (**a**) Experimental design. Samples for ChIP-Seq (using antibodies against PU.1, CEBPA and H3K27Ac) and gene expression microarray analysis were collected after 2 h incubation in the presence of OHT for two selected clonal lines, E1 (EV) and P2 (PuER). Samples for E1 and P2 were designated PU.1− and PU.1+, respectively. (**b**) New and stronger PU.1-bound regions can be detected after induction of wild-type PU.1. The genome browser screenshots show a comparison of PU.1-binding profiles between PU.1− and PU.1+ on the *Cd14* (upper) and *Elf1* (bottom) gene loci. A new peak at *Cd14* locus and a higher peak at *Elf1* locus can be detected following wild-type Pu.1 induction. (**c**) Global PU.1-binding site comparison between PU.1− and PU.1+ conditions. (**d**) Read count comparison between PU.1− and PU.1+ conditions, plotted as log_2_ of normalised read counts (per million reads) at PU.1 peaks, is shown. Line corresponding to direct correlation (no change) is depicted. (**e**) Global PU.1-binding site comparison between PU.1− and PU.1+ conditions and a published macrophage dataset (GSM537983). Strong overlapping between PU.1+ and macrophage datasets can be observed.

We next utilised this model to investigate the immediate changes in transcriptional control that accompany Pu.1-driven exit out of leukaemic proliferation. PU.1 primarily acts as a transcriptional activator, and can function in concert with CEBPA.^44, 45^ We therefore generated genome-wide ChIP-Seq data for the TFs PU.1 and CEBPA, and the H3K27Ac histone mark associated with transcriptional gene activation, at time 0 and 2 h after incubation in 100 nM OHT, in clonal samples for EV and PuER (henceforth designated PU.1− and PU.1+, respectively). Finally, RNA for gene expression microarrays was prepared from the same experiment, to link alterations in TF occupancy and/or histone modification levels with transcriptional changes (Figure 2a).

ChIP-qPCR assessment at the -14 Upstream Regulatory Element Pu.1-binding site revealed PU.1 binding in both EV and PuER clones, but with almost doubling of the enrichment in the PuER clone following OHT induction (Supplementary Figure 3a). This indicates that despite mutation of the DNA-binding domain, mutant PU.1 protein still possesses DNA-binding ability, although its functionality is likely to be impaired because we now show that it is unable to transactivate known PU.1 target gene promoters (Supplementary Figures 3b and c).

ChIP-Seq analysis with and without PU.1 induction revealed examples of both new PU.1-binding events, and increased binding at previously bound peaks (Figure 2b and Supplementary Figure 3d), with a total of 5283 PU.1 peaks in the absence of wild-type PU.1, increasing to 15 675 following induction, including 12 695 not previously detected (Figure 2c). Quantitative assessment of read counts per region, revealed a mean 7.5-fold increase in coverage score (Figure 2d) across all regions, indicating a generalised increase in PU.1 binding. Finally, comparing the peak regions bound by mutant and/or wild-type PU.1 with previously published data for PU.1 binding in primary macrophages,^36^ showed a significant overlap of binding peaks especially for wild-type PU.1 (Figure 2e).

This analysis demonstrates that although mutant PU.1 protein remains able to bind DNA, there is an increase in both the number and height of the peaks following wild-type PU.1 induction, with this binding profile showing substantial similarity to that in macrophages. As induction of wild-type PU.1 can overcome the differentiation block of X18.1.1 cells, the changes in PU.1 binding are presumably responsible for the initiation of a transcriptional programme that can exit from the leukaemic state into normal monocytic differentiation.

### Induction of wild-type PU.1 increases histone acetylation predominantly at enhancers

**Figure 3:**
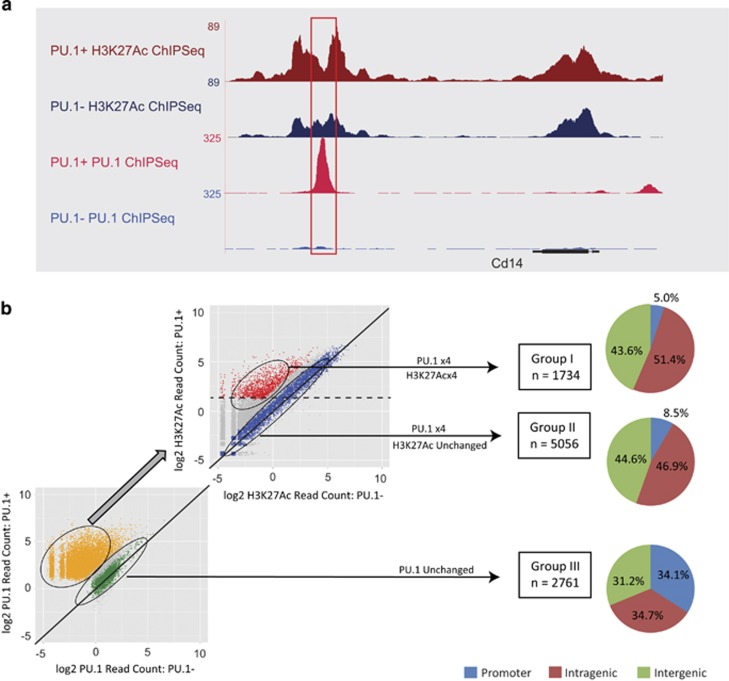
Integration of PU.1 and H3K27Ac ChIP-Seq binding profiles. (**a**) Genome browser screen shots show increased signal for H3K27Ac histone mark (upper two tracks) on a new PU.1 peak (bottom two tracks) detected at the *Cd14* locus for PU.1+ (red) and PU.1− (blue) conditions. (**b**) Three different groups of PU.1-bound regions were defined. Firstly, read count comparison (log2 normalised read count) for regions bound by PU.1 in PU.1− and PU.1+ conditions were plotted. Regions with no change in PU.1 binding defined Group III (2761 regions shown in green). Then, H3K27Ac read count comparison in PU.1− and PU.1+ conditions for regions with ⩾fourfold change in PU.1 comparison (shown in yellow) was plotted. Regions with no change in H3K27Ac enrichment defined Group II (5056 regions shown in blue). Regions with ⩾50 counts integrated Group I (1734 regions, shown in red). Line corresponding to direct correlation (no change) is shown in both plots. Threshold corresponding to 50 counts in H3K27Ac read count comparison plot is depicted with dashed line. Regions were then associated to genes and distribution of promoter, intragenic and intergenic localisation of the regions for each of the three groups is shown.

Comparison across all PU.1-bound regions revealed a positive correlation between changes in PU.1 binding and those of H3K27Ac (Pearson correlation 0.37, *P*<0.0001) (Supplementary Figure 4a), indicating that the induction of wild-type PU.1 leads to co-localisation of histone modifications associated with increased transcriptional activity at many of its target sites (Figure 3a). On the basis of these results, we defined a set of peaks representing functional PU.1 binding (Group I, *n*=1734) defined by (i) ⩾fourfold increase in PU.1 read count, (ii) ⩾fourfold increase in H3K27Ac read count and (iii) H3K27Ac read number ⩾50 per region. For comparison, we looked at peaks with ⩾fourfold increase in PU.1 but unchanged H3K27Ac (Group II, *n*=5056), and those with no change in PU.1 (Group III, *n*=2761) (Figure 3b). Of note, *de novo* motif discovery demonstrated that ETS motifs previously reported to be bound specifically by Pu.1 were more highly enriched in Group I than Group II peaks (Supplementary Figure 5).

Analysis of the genomic distribution of PU.1-binding peaks revealed a marked enrichment of the peaks with increased read counts for intra- and intergenic localisation (95.0% and 91.5% for Groups I and II, respectively) compared with those with no read-count change (65.9 for Group III). Even though the association of peaks to genes is complicated by the increased recognition of long range promoter-enhancer interactions,^46^ our data are consistent with tissue-specific functions of PU.1 being largely mediated by enhancer region binding,^36^ and shows that these binding events may have an important role in the phenotypic switch to monocytic maturation.

### PU.1 recruitment coupled with increased histone acetylation induces gene expression and activates a monocyte/macrophage transcriptional programme

**Figure 4:**
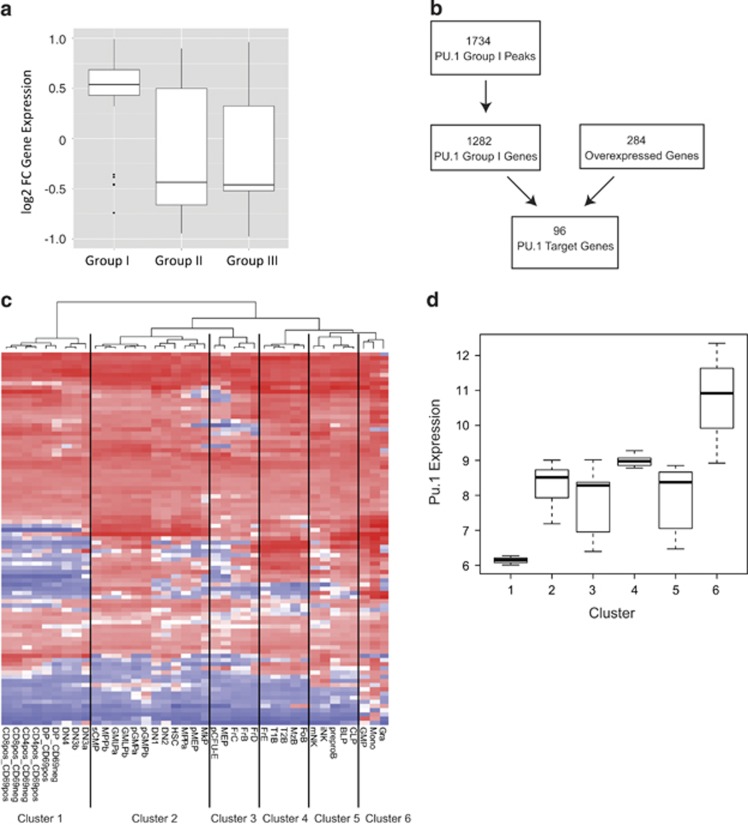
Increased PU.1 binding and increased H3K27Ac enrichment define a set of target genes that can be used as classifier for normal haematopoietic cells. (**a**) Greatest upregulation can be detected in genes with increased PU.1 binding and increased H3K27Ac enrichment. Plot shows overall changes in expression (log_2_FC) of genes from Groups I, II and III with a significant variation of expression following wild-type PU.1 induction. (**b**) Peaks in Group I (1734 regions) were associated to genes (1282 Group I genes) and intersected with genes that showed increased expression on microarray analysis following 2-h PU.1 induction (284 genes) to obtain high confidence PU.1 target genes (96 genes). (**c**) Unsupervised hierarchical clustering of gene expression from phenotypically defined murine haematopoietic cell types^39^ on basis of 89 of 96 PU.1 target genes expression present in dataset. Defined clusters are indicated. Out of six defined clusters, cluster 6 (integrated by GMPs, granulocytes and monocytes) present highest levels of *Pu.1* (**d**). (Full phenotypic definitions of cell types may be found in Seita *et al.*^39^)

We next explored the early transcriptional consequences of the set of genomic regions associated with increased PU.1 binding and H3K27 acetylation. Unsupervised hierarchical clustering of gene expression profiles after only 2 h of induction, separated the samples into two groups corresponding to the presence or absence of wild-type PU.1 (Supplementary Figure 4b). We associated the PU.1 peak sets to genes, and compared expression profiles before and after OHT induction. We calculated the fold-change for those genes with a false discovery rate-adjusted *P*-value of <0.05 and compared the overall change of expression for each of the three groups previously defined. Not only were many of the genes that changed expression associated with Group I peaks (96 out of 284), but also genes associated to Group I showed the most increased expression following PU.1 induction, compared with Group II and Group III peaks (fold-change 1.76 vs 0.82, *P*<0.0001 and 1.76 vs 0.81, *P*<0.0001, respectively) (Figure 4a). This confirms that even at the early time-point of 2 h, recruitment of wild-type PU.1 accompanied by increased H3K27 acetylation initiates a PU.1-driven transcriptional programme.

To further examine the relationship between PU.1-binding events and gene expression, we took the 96 gene overlap of the 1282 genes mapped from the PU.1 Group I peaks with the 284 genes that showed increased expression following PU.1 induction (Figure 4b). Gene ontology analysis of this 96 PU.1 target gene list revealed overrepresented biological functions to be apoptosis and humoral immune response, while enrichment analysis showed strongest overlaps with expression profiles specific to macrophages or dendritic cells (Supplementary Table 2).

Finally, we used the PU.1 target genes to perform unsupervised hierarchical clustering of gene expression profiles for 39 murine haematopoietic cell types^39^ (Figure 4c, Supplementary Figure 6). This produced six major clusters, of which Cluster 6 was characterised by the highest expression levels of Pu.1, and consisted exclusively of myeloid and monocytic cells. Intermediate clusters showed a B-cell bias in those with higher Pu.1 expression, whereas Cluster 1 consisting exclusively of T-cell subtypes had the lowest Pu.1 expression levels (Figure 4d). This is consistent with the known functions of PU.1 in myeloid and B-cell differentiation, and validates our gene list as representing physiological PU.1-driven pathways.

### Restoring functional PU.1 causes reorganisation of CEBPA binding

**Figure 5:**
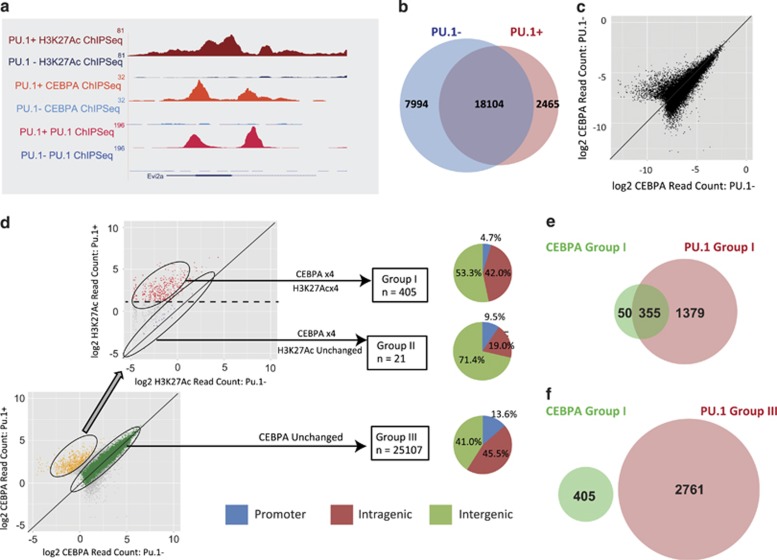
Regions with increased CEBPA binding and increased H3K27Ac enrichment co-localise with regions with increased PU.1 binding and increased H3K27Ac enrichment following wild-type PU.1 induction. (**a**) Genome browser screenshots show new regions bound by CEBPA (middle two tracks) at regions with increased signal for H3K27Ac histone mark (upper two tracks) on new PU.1 peaks (bottom two tracks) detected at the *Evi2a* locus for PU.1+ (red) and PU.1− (blue) conditions. (**b**) Global CEBPA-binding site comparison between PU.1− and PU.1+ conditions. (**c**) Read count comparison between PU.1− and PU.1+ conditions, plotted as log_2_ of normalised read counts (per million reads) at CEBPA-bound regions, is shown. Line corresponding to direct correlation (no change) is depicted. (**d**) Three different groups of CEBPA-bound regions were defined. Firstly, read count comparison for regions bound by CEBPA in PU.1− and PU.1+ conditions were plotted (log2 normalised read count). Regions with no change in CEBPA binding defined Group III (25107 regions shown in green). Then, H3K27Ac read count comparison in PU.1− and PU.1+ conditions for regions with ⩾fourfold change in CEBPA comparison (shown in yellow) was plotted. Regions with no change in H3K27Ac enrichment defined Group II (21 regions shown in blue). Regions with ⩾50 counts integrated Group I (405 regions, shown in red). Line corresponding to direct correlation (no change) is shown in both plots. Threshold corresponding to 50 counts is shown in H3K27Ac read count comparison plot by dashed line. Regions were then associated to genes and distribution of promoter, intragenic and intergenic localisation of the regions for each of the three groups is shown. (**e**) Global binding site comparison between CEBPA Group I peaks and PU.1 Group I peaks. (**f**) Global binding site comparison between CEBPA Group I peaks and PU.1 Group III peaks.

On investigating the relationship between PU.1 and CEBPA (Figure 5a), ChIP-Seq data showed that the overall peak numbers were much higher for CEBPA with 26 098 peaks present before and 20 569 peaks present after wild-type PU.1 induction (Figure 5b), with mean read count over all regions essentially unchanged. However, 2465 regions were called as peaks only after PU.1 induction, and these showed a mean 2.3-fold increase in read count, suggesting a discrete group of CEBPA-binding events enhanced by the presence of active PU.1 (Figure 5c).

To define relevant CEBPA peaks, we used the same methodology as above and defined a set of 405 Group I peaks defined by increased CEBPA and increased H3K27Ac read count (Figure 5d). Again, control groups representing CEBPA increase without H3K27Ac change (Group II, *n*=21) and unchanged CEBPA binding (Group III, *n*=25 107) were selected for comparison. The small number of Group II peaks indicates that the vast majority of CEBPA peaks with increased binding after PU.1 induction were associated with H3K27Ac change, and are likely to be functionally relevant for gene transcription (Figure 5d).

As with PU.1, the Group I CEBPA peaks were relatively enriched at intra- and intergenic regions (95.3%), when compared with Group II and Group III (90.4% and 86.5% respectively) (Figure 5d). Comparing the effect on expression from the genes mapped from these peak sets, again revealed that Group I genes showed significantly higher expression after PU.1 induction (2.1-fold) compared with those from Group III which showed no fold-change. There were no genes mapped from the small number of Group II CEBPA peaks that corresponded to genes with altered expression on the array (Supplementary Figure 4c). These results show that although there is widespread CEBPA binding before PU.1 induction, a small subset of binding regions are induced by wild-type PU.1 binding, and may contribute to the transcriptional programme.

*De novo* motif analysis of the PU.1 peak sequences showed CEBPA motifs overrepresented in all three groups of PU.1 peaks defined in the previous section. However, the percentage of regions with CEBPA motifs was higher in Group I PU.1 peaks (36.0%), compared with either Group II (26.4%) or Group III (26.1%) (Supplementary Table 3). This observation suggested that either (i) Group I PU.1 peaks preferentially bind to regions already ‘bookmarked' by prior CEBPA binding or, alternatively, (ii) Group I PU.1 peaks bind to new genomic regions, and themselves act as markers for new CEBPA binding.

Co-binding between peak sets showed that 355/1734 (20.5%) of the Group I PU.1 peaks were at sites of pre-existing CEBPA, a proportion no higher than that of the (presumably non-functional) Group II and III PU.1 peaks (1331/5056 (26.33%) and 1517/2761 (54.94%), respectively). This does not support a significant role for CEBPA in marking sites for subsequent functional PU.1 binding.

We then investigated the alternative possibility that Group I PU.1 peaks bind to new genomic regions and recruit subsequent CEBPA binding. Of the 405 Group I CEBPA peaks identified, 355 (87.7%) were bound at Group I PU.1 peaks; in comparison, none (0.0%) of them were co-bound by PU.1 Group III peaks (Figures 5e and f). This suggests a role for wild-type PU.1 in making new regions available for subsequent functional binding of CEBPA, but not the converse.

### PU.1 target genes can predict human AML subtypes regulated by PU.1 dysfunction

**Figure 6:**
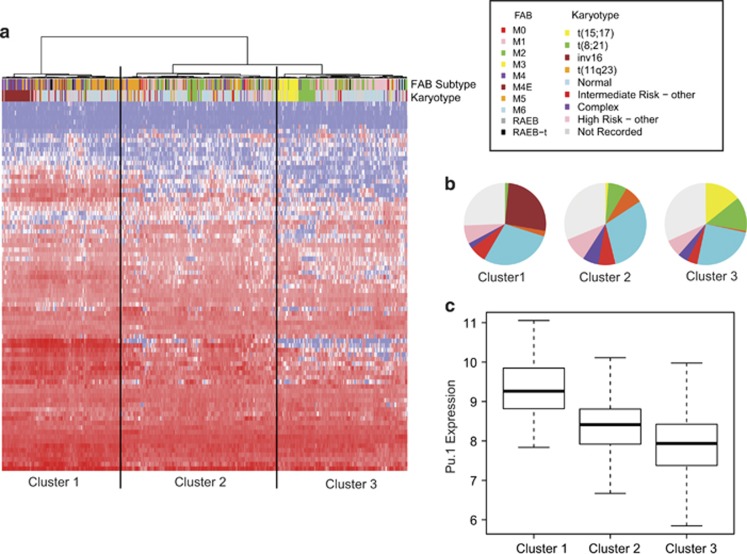
Increased Pu.1 binding and increased H3K27Ac enrichment define a set of target genes that can be used to classify primary AML samples. (**a**) Unsupervised hierarchical clustering of gene expression from a large well-annotated AML dataset^40^ on basis of expression of 81 of 96 PU.1 target genes present in dataset (defined in Figure 4). Colourings for FAB subtype and karyotype classifications are shown and defined clusters are indicated. Karyotype proportions (**b**) and PU.1 expression levels (**c**) in each of the three clusters are shown.

To assess the extent to which this murine model corresponds with human AML, we used the PU.1 target gene set to perform unsupervised hierarchical clustering of a set of 461 primary human AML samples with genome-wide expression data^40^ (Figure 6a, Supplementary Figure 7). This generated three clusters, of which Cluster 3 showed reduced levels of *PU.1* expression, and contained 21/23 (91.3%) of the t(15;17) translocations and 20/35 (57.1%) of the t(8;21) translocations (Figures 6b and c). This may represent AML samples driven in part by *PU.1* underexpression and/or dysfunction. Cluster 1 samples had the highest levels of *PU.1* expression, and were predominantly M4/M5 FAB classification (myelomonocytic/monoblastic), suggesting a separate class of cases where high *PU.1* levels direct a degree of monocytic differentiation, but alternative mechanisms are responsible for the leukaemia phenotype (Figure 6c). Taken together, this analysis suggests that the murine model is not only relevant for defining PU.1 target genes that may be involved in the leukaemic phenotype of t(15;17) and t(8;21) AML, but also that it may be exploited to identify novel human AML subtypes.

## Discussion

### Relevance of Pu.1 mutations

Mutations in the Pu.1 DNA-binding domain are common in radiation-induced murine leukaemia,^19^ although not in human AML.^47^ Within the DNA-binding domain, R235 is highly conserved across species, and structural studies have shown that it has a crucial role in DNA binding at the GGAA core of the Ets motif, leading to the prediction that DNA binding would be impossible in its mutated state.^3^ Unexpectedly, our ChIP data clearly demonstrate the DNA-binding capacity of this mutant PU.1 protein. However, a prominent overlap with PU.1-binding profiles in macrophages was only observed following the expression of wild-type PU.1, suggesting that it is the limited binding ability of mutant PU.1 rather than complete absence which causes differentiation block. Consistent with this notion, the expression of wild-type PU.1 increased histone acetylation at a subset of newly bound regions, and the concomitant upregulation of neighbouring genes identified a gene set associated with monocyte differentiation.

### Effect of PU.1 induction on CEBPA distribution

It is well recognised that many myeloid gene regulatory elements are simultaneously activated by PU.1 and CEBPA.^44, 45^ Although CEBPA has been reported to inhibit PU.1 function by displacing the co-activator c-Jun from binding to the B3/B4 region of the DNA-binding domain,^48^ positive interactions between PU.1 and CEBPA are exemplified by CEBPA-mediated activation of Pu.1 expression by binding to the *PU.1* promoter^49^ and Upstream Regulatory Element enhancer sites,^50, 51^ and PU.1 regulation of CEBPA expression by binding at the *CEBPA* +37 enhancer region.^52^ Given this complex interplay, the ratio of PU.1 to CEBPA has been argued to determine whether cell fate is predominantly monocytic or granulocytic^9, 21^ with modelling predicting a bistable differentiation potential between the two lineages when both TFs are highly expressed.^53^

In our system, CEBPA binding was widespread both before and after wild-type PU.1 induction, but we were able to identify a group likely to represent new and functionally significant events. As with PU.1, the relative depletion of these peaks in non-enhancer regions suggests cell-type-specific functionality. The finding that 87.7% of these were also bound by PU.1 Group I peaks indicates that this discrete subgroup of CEBPA-binding sites are ‘bookmarked' by the presence of PU.1 to facilitate subsequent binding.

It therefore appears that a proportion of normal CEBPA-binding events are impaired by the absence of PU.1 in the leukaemic phase, which may contribute to the differentiation block seen in PU.1-dysregulated leukaemias. Our findings are consistent with a non-leukaemic PuER model of Pu.1^-/-^ cells, where a relatively small group of CEBPB peaks were observed after PU.1 induction with strong enrichment for PU.1 motifs, and were co-bound by PU.1 in over 75% of cases.^36^ CEBPA co-localisation to existing PU.1 peaks was also noted in a recent study of RUNX1-ETO knockdown in a t(8;21) AML cell line.^54^

### ‘Functional' PU.1 binding induces monocytic/macrophage differentiation to overcome the leukaemia phenotype

PuER induction caused both quantitative and qualitative differences in binding between wild-ype and mutant PU.1, and induced PU.1-binding sites with a concomitant increase in histone H3K27 acetylation were primarily localised to enhancers. Cell-type-specific binding of PU.1 in non-leukaemic cells had previously been reported to largely be enhancer-specific,^36^ and associated with elevated H3K27 acetylation.^55^ These observations are therefore consistent with the notion that introducing wild-type PU.1 into this leukaemic model activates a transcriptional programme related to normal cell maturation, overcoming the differentiation block. Our findings not only provide a clearer understanding of the mechanism for the role of PU.1 suppression in AML, but also identify activation of PU.1 as a potential therapeutic target for differentiation therapy in human AML.

Although *PU.1* mutations are uncommon in human AML,^25, 26, 27, 47, 56^ upstream mechanisms causing reduced *PU.1* expression and function are well described for the fusion genes RUNX1-ETO^11^ and PML-RARA,^28, 57^ as well as by FLT3-ITD mutations.^58, 59^ Analysis of primary AML samples using the set of 96 PU.1 target genes, clustered the majority of RUNX1-ETO and PML-RARA samples together in a group characterised by low *PU.1* expression. The presence of other AML cases in this cluster suggests that other as yet unidentified pathways may converge on *PU.1* under-expression, giving rise to a common molecular defect.

Although genome reprogramming technologies are already showing potential in manipulating TF expression to alter phenotype,^60^ it is difficult to see at present how genetic targeting will achieve the efficiency required to target all leukaemic cells within a patient for effective differentiation. As ATRA has proved in PML-RARA-driven AML,^61^ pharmacological interventions on the other hand represent a potent method of overcoming differentiation block and treating the leukaemia. Interestingly, PU.1 induction has recently been demonstrated by intravenous cytokine activation,^62^ and it may be that a small molecule-based therapeutic to upregulate PU.1 and alter downstream target expression will have clinical potential to treat PU.1-dysregulated AML.
