# Supplementary material for: Genome-scale definition of the transcriptional programme associated with compromised PU.1 activity in acute myeloid leukaemia
Source: Leukemia. 2015 Jul 21;30(1):14–23. doi: 10.1038/leu.2015.172 (PMC4705427; doi:10.1038/leu.2015.172)
Supplement: Supplementary Information [file leu2015172x1.pdf]

Genome-scale definition of the transcriptional programme associated with compromised PU.1 activity in Acute Myeloid Leukaemia

Jonathan I. Sive, Silvia Basilico, Rebecca Hannah, Sarah J. Kinston, Fernando J. Calero-Nieto, Berthold Göttgens

Supplementary Methods

FACS and cell proliferation assays

Flow cytometry was performed on a BD LSRFortessa cell analyser using the following antibodies: CD117 (c-kit) (Biolegend, 105826), CD34 (BD Pharmingen, 553733), b220 (BD Pharmingen, 553085), CD3 (BD Pharmingen, 553059), V500 Streptavidin (BD Horizon, 561419), Ly-6A (Sca-1) (Biolegend, 122520) and Ly-6G/Ly-6C (Gr-1) (BD Pharmingen, 553128). Cell proliferation was assayed by counting live cells following Trypan Blue exclusion.

Transactivation assays

293T cells were transfected with 1 µg 'luciferase' construct in combination with 3 µg pcDNA3-PU.1 (generous gift from Dr J. Frampton,Birmingham, UK), pcDNA3-mutant PU.1 or the empty vector pcDNA3 as control using ProFection Mammalian TransfectionSystem-Calcium Phosphate (Promega). Each transfection and transactivation was performed on three different days in triplicate. To obtain the pcDNA3-mutant PU.1 construct, a fragment XcmI-

SfiI of pcDNA3-PU.1 vector was replaced by a synthesised XcmI-SfiI fragment (Life Technologies) containing the mutant PU.1 sequence present in the parental line X18.1.1.

Immunoblotting

Protein lysate in RIPA buffer (50mM Tris pH7.4, 150mM NaCl, 1% NP40, 0.5% Na deoxycholate, 0.1% SDS) was run on a 8% SDS polyacrylamide gel, and transferred to PVDF membrane by overnight wet blotting. Membranes were probed using primary antibody against PU.1 (Santa Cruz, sc352x).

Gene expression analysis

Total RNA was isolated using Tri-Reagent (Sigma-Aldrich) according to the manufacturer's instructions. Contaminating genomic DNA was removed by DNAseI treatment with Turbo DNA free (Applied Biosystems/Ambion). Total RNA (400 ng) was used for cDNA synthesis, using SuperScript® III First-Strand Synthesis System for RT-PCR and random hexamers (Invitrogen). Total and wild type *Pu.1* levels were measured by real-time PCR using specific primers (5' ATGCACGTCCTCGATACTCC 3'and 5' GCTGGGGACAAGGTTTGATA 3' for total Pu.1, 5' CCCGGATGTGCTTCCCTTAT 3' and 5' TCCAAGCCATCAGCTTCTCC 3',[1] , for wild type). Absolute expression was calculated from a standard curve obtained with ScaI-linearised pcDNA3-PU.1 vector. Mutant *Pu.1* levels were calculated as the difference between total and wild type *Pu.1* levels. Experiments were performed on 2 biological replicates.

1. Anderson MK, Weiss AH, Hernandez-Hoyos G, Dionne CJ, Rothenberg EV. Constitutive expression of PU.1 in fetal hematopoietic progenitors blocks T cell development at the pro-T cell stage. *Immunity* 2002; **16:** 285-296.

Supplementary Figure Legends

Supplementary Figure 1.

(a) Cell growth was specifically arrested in PuER line after 4 days of OHT incubation. Growth curves for Empty Vector and PuER bulk populations are shown during 4 days in presence (+ OHT) or absence (-OHT) of OHT. Mean and SEM for 2 different experiments (each one performed in triplicate) are shown.

(b) Restoration of wild type PU.1 induces upregulation of CD11b and F4/80 surface markers after 4 days, although compared to clonal lines a degree of leakage is seen in PuER bulk population. FACS results corresponding to CD11b and F4/80 surface expression in Empty Vector and PuER bulk populations are shown.

(c) Effect of restoration of wild type PU.1 on the cellular immunophenotype. FACS results corresponding to CD117, CD34, b220, CD3, Sca-1 and Gr-1 surface expression in two selected clonal lines, Empty Vector (E1) and PuER (P2), following 4 days in presence (solid area) or absence (transparent area) of OHT are shown.

Supplementary Figure 2

(a) Detection of stable integration of PuER. Genomic DNA was extracted from the X18.1.1 parental line, Empty Vector (EV) and PuER bulk populations and selected clonal lines (E1, E2, P1, P2) and used for detection of PuER by PCR. *Gata2* was used as a control.

(b) Absolute quantification of mRNA PU.1 levels. Total (black bars), wild type (grey bars) and mutant (white bars) mRNA levels of PU.1 were measured in the parental line X18.1.1, Empty Vector and PuER bulk populations and the 2 selected clonal lines, Empty Vector (E1)

and PuER (P2). For comparison, wild type PU.1 mRNA levels were also measured in the Raw monocytic/macrophage cell line. Mean and SEM for 2 different experiments (each one performed in triplicate) are shown. nd: not detected.

Supplementary Figure 3

(a) PU.1 is specifically recruited at higher levels at the -14 Upstream Regulatory Element (URE) of the *Pu.1* locus after restoration of fully functional PU.1. DNA recovery of ChIP material using an antibody against PU.1 was quantified by quantitative PCR at the -14 URE and at a negative control region within Chromosome 1 (Chr 1) with and without OHT induction for selected clonal lines corresponding to Empty Vector (E1) and PuER (P2). Although ChIP material could be detected with mutant PU.1, a much higher enrichment at this region is obtained after restoration of wild type PU.1.

(b,c) Transactivation of regulatory elements by wild type and mutant PU.1. To assess mutant functionality, transient co-transfections of 293T cells with luciferase reporter constructs containing Lyl1 promoter or Elf-1 -21 regulatory elements in combination with either pcDNA3-PU.1 (PU.1), pcDNA3-mutant PU.1 (mPU.1) or the empty vector pcDNA3 (control) were performed. Values are expressed relative to the control, pcDNA3. Mean and SEM for 3 independent transfections (each one performed in triplicate) are shown. As a control, wild type and mutant PU.1 proteins were detected using an antibody against PU.1. Statistical comparison was performed by one-way analysis of variance followed by post-hoc analysis with the Bonferroni test for selected pairs of columns. ***$P < 0.001$.

(d) The genome browser screenshot shows a comparison of PU.1 binding profiles on the *Cd14* gene locus between the selected clonal lines corresponding to Empty Vector (E1) and PuER (P2) in the presence or absence of OHT induction.

Supplementary Figure 4

(a) Change in normalised read counts in PU.1- and PU.1+ conditions for regions bound by PU.1 were calculated. Similarly, changes in H3K27Ac read counts for the same regions were also calculated. Log2 of the values for each region were plotted to investigate the correlation. Line corresponding to direct correlation is depicted.

(b) Unsupervised hierarchical clustering of microarray gene expression data

(c) Greatest upregulation can be detected in genes with increased CEBPA binding and increased H3K27Ac enrichment. Plot shows overall changes in expression (log_2FC) of genes from CEBPA Groups I and III with a significant variation of expression following wild type PU.1 induction.

Supplementary Figure 5

Top three motifs from de novo motif analysis for PuER and EV Pu.1 peaks. The HOMER platform (http://homer.salk.edu/homer/motif/) was used to identify the most highly enriched sequence motif in the two respective peak sets, which were then compared to known motifs present in the Jaspar motif database (http://jaspar.genereg.net/). This analysis showed that the highest-scoring motifs matched to Ets consensus sites in both cases. However, for the PuER sample (panel A), the top hits matched specifically to the recognised Pu.1 consensus binding

site (PB0058.1_Sfpi1_1/Jaspar motif). By contrast, for the empty-vector control sample (panel B), the top hits were reported as ETS1(ETS)/Jurkat-ETS1-ChIPSeq/Homer. This observation is consistent with a difference in transcriptional function of wild-type versus mutant Pu.1 protein.

Supplementary Figure 6

Enlarged version of Figure 4c showing clustering and gene names. Unsupervised gene clustering of expression from phenotypically defined murine haematopoietic cell types, classified on basis of expression of PU.1 target gene set. Clusters derived from analysis are indicated. (Full phenotypic definitions of cell types may be found in reference 39 of manuscript)

Supplementary Figure 7

Enlarged version of Figure 6a showing unsupervised clustering and gene names. Heatmap showing expression of primary AML samples classified using PU.1 target gene set. Clusters derived from analysis are indicated.

Supplementary Table 1 - Primers

Supplementary Table 2. Gene ontology analysis (Enrichr)

Supplementary Table 3. Peak set *de novo* motif analysis

Supplementary Figure 1

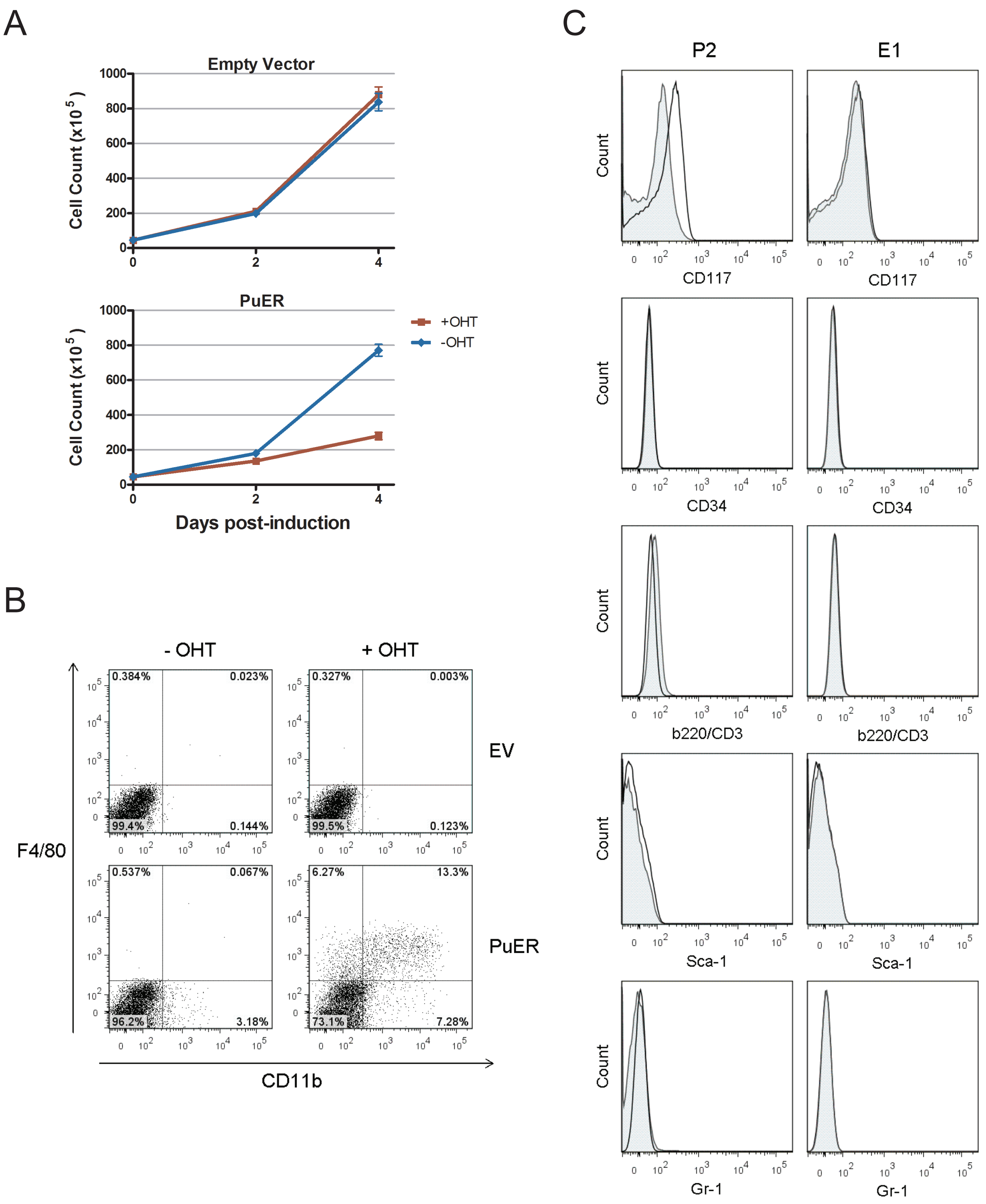

Supplementary Figure 2

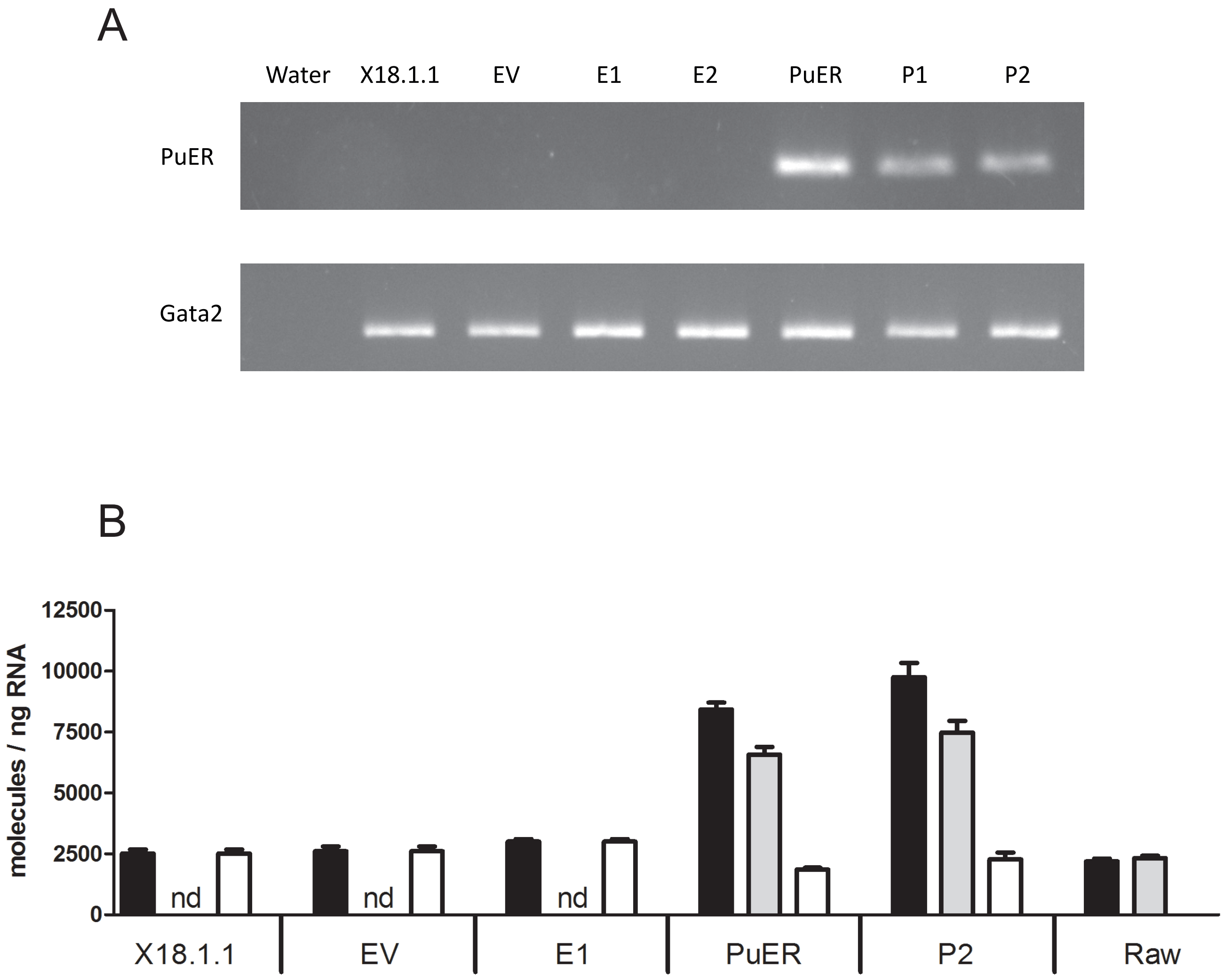

Supplementary Figure 3

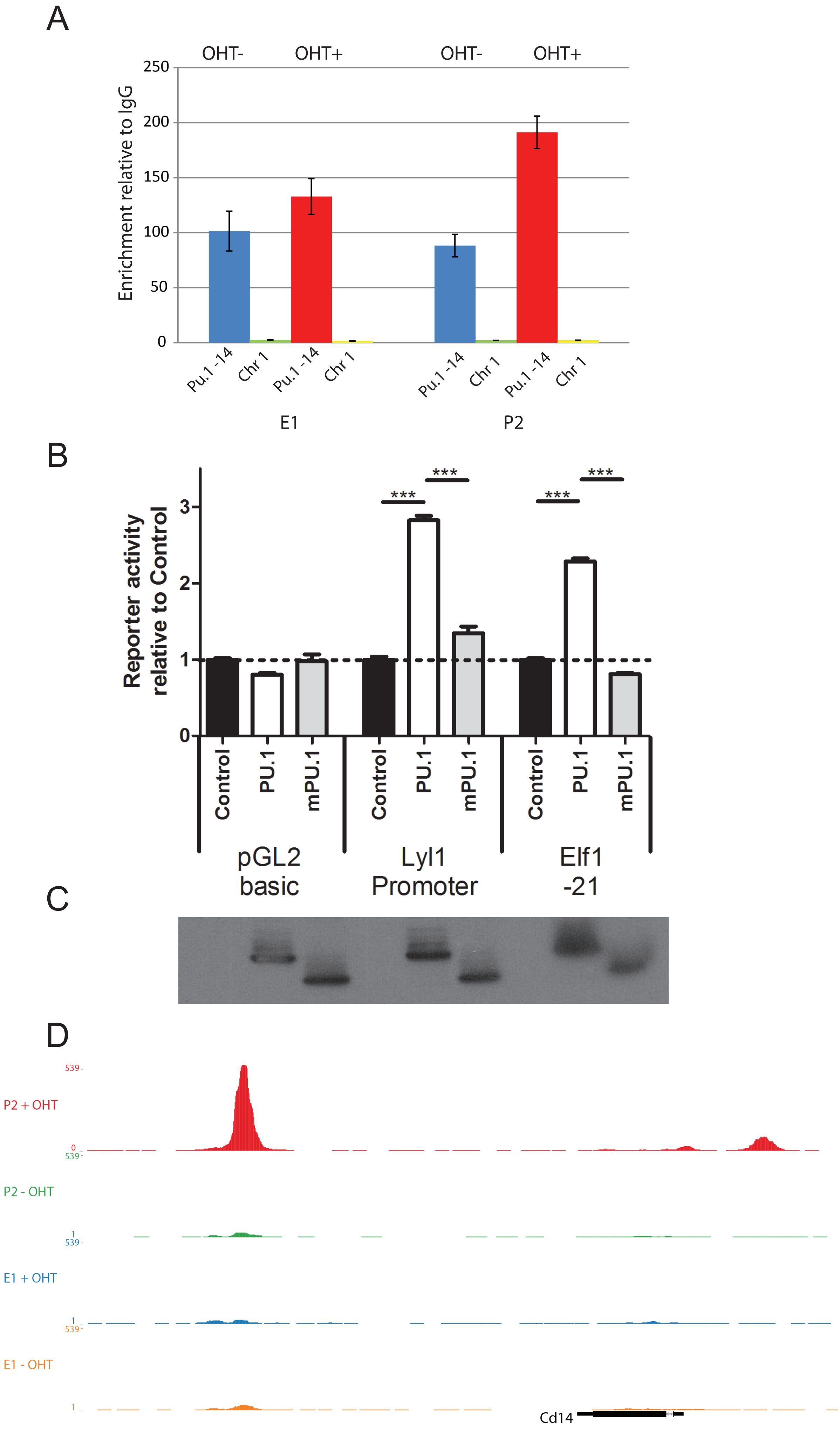

Supplementary Figure 4

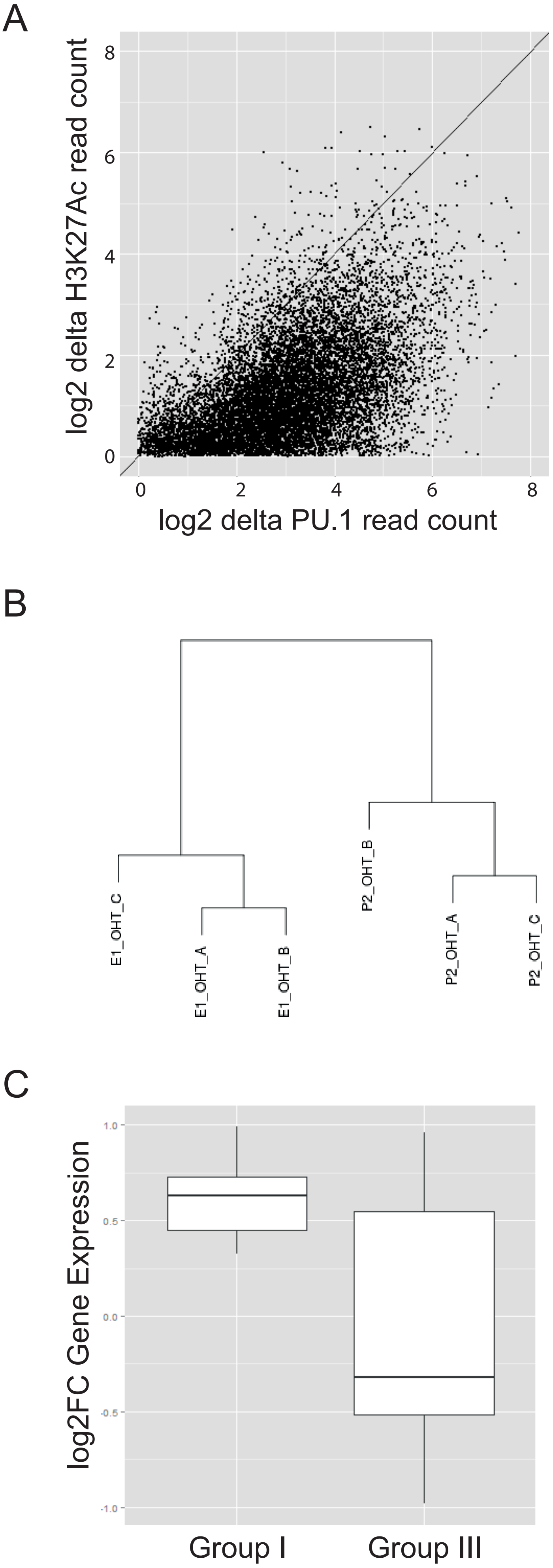

Supplementary Figure 5

A

Rank	Motif	P-value	log P-pvalue	% of Targets	% of Background	STD(Bg STD)	Best Match/Details	Motif File
1	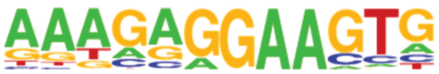	1e-6092	-1.403e+04	57.98%	6.97%	38.9bp (73.8bp)	PB0058.1_Sfpi1_1/Jaspar More Information | Similar Motifs Found	motif file (matrix)
2	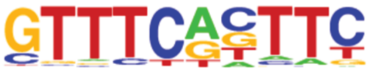	1e-1283	-2.956e+03	17.73%	2.83%	51.5bp (68.3bp)	PU.1-IRF/Bcell-PU.1-ChIP-Seq/Homer More Information | Similar Motifs Found	motif file (matrix)
3	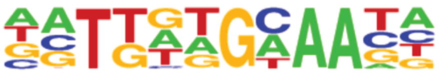	1e-935	-2.155e+03	22.92%	6.60%	49.0bp (62.9bp)	MF0006.1_bZIP_cEBP-like_subclass/Jaspar More Information | Similar Motifs Found	motif file (matrix)

B

Rank	Motif	P-value	log P-pvalue	% of Targets	% of Background	STD(Bg STD)	Best Match/Details	Motif File
1	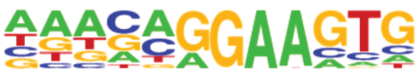	1e-1560	-3.593e+03	44.56%	4.96%	41.8bp (63.2bp)	ETS1(ETS)/Jurkat-ETS1-ChIP-Seq/Homer More Information | Similar Motifs Found	motif file (matrix)
2	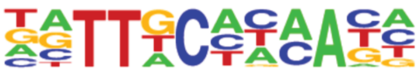	1e-674	-1.553e+03	33.86%	7.38%	46.6bp (63.5bp)	MF0006.1_bZIP_cEBP-like_subclass/Jaspar More Information | Similar Motifs Found	motif file (matrix)
3	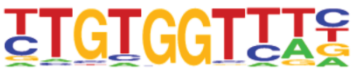	1e-300	-6.926e+02	23.02%	6.93%	48.4bp (62.5bp)	RUNX(Runt)/HPC7-Runx1-ChIP-Seq/Homer More Information | Similar Motifs Found	motif file (matrix)

Supplementary Figure 6

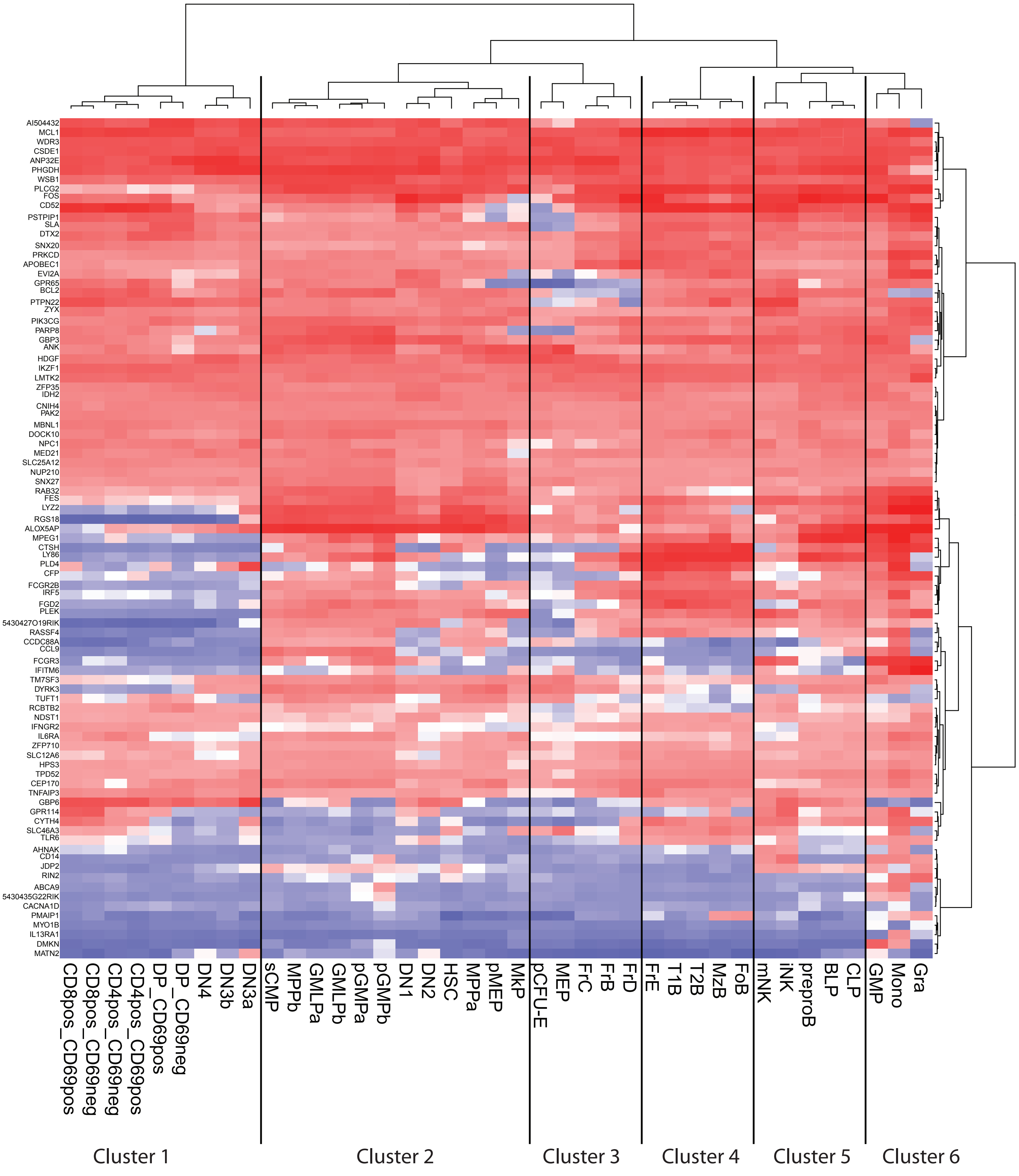

Supplementary Figure 7

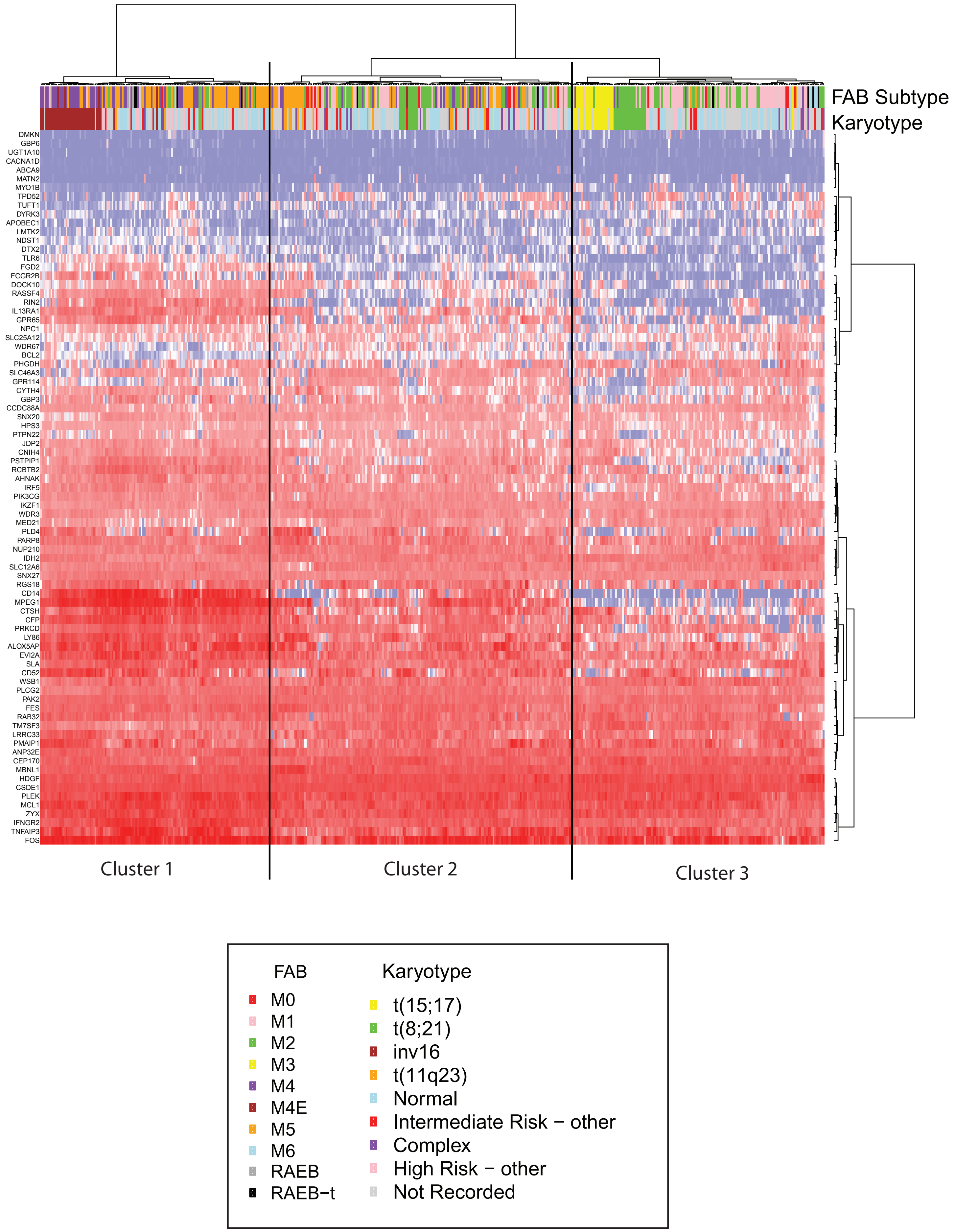

Supplementary Table 1 - Primers

PCR Primers

	Forward	Reverse
PuER	CTTCCAGTTCTCGTCCAAGC	CCTGAAGCACCCATTTCATT
GATA2	GCTGGACATCTTCCGATTCCGGGT	GGAACGCCAACGGGGAC
Pu.1 Exon 1	GTAGACGGATGGGTGGTGTG	GGCATGCTCTAAGGAAGCCA
Pu.1 Exon 2	GGGTTAGTGGGCAGATGGTC	TCCCCAAGGACCAGACAAGA
Pu.1 Exon 3	TTAGGAAACCTCTCTGCGCC	AAGGTGTGGGTCCCTAAGGT
Pu.1 Exon 4	ATTCCTAGTGCACTGCCAGG	GCATCCACAAACACGCATGT
Pu.1 Exon 5	GTGTGCACTTGAGTGGGAGT	CTAGGGCCCAGCTTTGGAAA
Pu.1 cds	CTGAGCCCTGCGTCTGAC	GGGCGACGGGTTAATGCTAT

ChIP qPCR Primers

	Forward	Reverse
Pu.1 -14	GCTGTTGGCGTTTTGCAAT	GGCCGGTGCCTGAGAAA
Chromosome 1	CATAGATGAAGCTGCCACATAGGT	GTGGGCAAGGACAAAGCATTA

Sequencing Primers

T7 Prom fwd	TAATACGACTCACTATAGGG
SP6 Prom rev	ATTTAGGTGACACTATAG

Supplementary Table 2. Gene ontology analysis (Enrichr)

GO Biological Process

Term	Overlap	P-value	Z-score	Combined Score	Genes
immune response-activating signal transduction (GO:0002757)	9/440	<0.01	-3.54	11.71	TNFAIP3;PTPN22;BCL2;CD14; TLR6;PLCG2;FOS;PAK2;PRKCD
activation of immune response (GO:0002253)	10/487	<0.01	-3.46	11.58	TNFAIP3;PTPN22;CFP;BCL2; CD14;TLR6;PLCG2;FOS;PAK2 ;PRKCD
defense response to other organism (GO:0098542)	9/328	<0.01	-2.37	8.45	CFP;GBP6;BCL2;APOBEC1; PMAIP1;TLR6;GBP3;PRKCD; IRF5
regulation of cell activation (GO:0050865)	9/420	<0.01	-2.52	8.33	IL13RA1;TNFAIP3;FES; PTPN22;IKZF1;BCL2;PLEK; PAK2;PRKCD
response to other organism (GO:0051707)	10/462	<0.01	-2.38	8.05	GBP6;CFP;BCL2;APOBEC1 ;PMAIP1;TLR6;GBP3;IFNGR2; PRKCD;IRF5
response to metal ion (GO:0010038)	7/255	<0.01	-2.38	7.87	NPC1;BCL2;ALOX5AP;CD14; APOBEC1;SLC25A12;FOS
positive regulation of apoptotic signalling pathway (GO:2001235)	6/168	<0.01	-2.35	7.76	PMAIP1;MCL1;PAK2;PRKCD ;CTSH;BCL2
inflammatory response (GO:0006954)	8/376	<0.01	-2.45	7.69	PIK3CG;LY86;CD14;TLR6; TNFAIP3;NDST1;FOS;PSTPIP1
leukocyte activation (GO:0045321)	8/373	<0.01	-2.43	7.61	PTPN22;TPD52;PIK3CG;IKZF1 ;BCL2;TLR6;PLCG2;PRKCD
response to inorganic substance (GO:0010035)	8/370	<0.01	-2.40	7.53	NPC1;BCL2;ALOX5AP;CD14; APOBEC1;TNFAIP3;SLC25A12; FOS

Mouse Gene Atlas

Term	Overlap	P-value	Z-score	Combined Score	Genes
macrophage_bone_marrow_6hr_LPS	14/730	0.00	-2.24	15.40	RASSF4;CCL9;SNX20;TPD52;CFP; FCGR3;PLEK;FCGR2B;CD14;TLR6; IFNGR2;CYTH4;GM885;DTX2
macrophage_bone_marrow_2hr_LPS	6/365	0.02	-1.98	7.80	5430427O19RIK;NUP210;CD14; JDP2;CCL9;EVI2A
dendritic_cells_myeloid_CD8a-	6/188	0.01	-1.49	7.10	PMAIP1;ABCA9;SLC46A3; CCDC88A;CTSH;GPR114
RAW_264_7	6/222	0.01	-1.61	6.75	IL13RA1;ZFP710;APOBEC1;AHNAK; CYTH4;IL6RA
macrophage_peri_LPS_thio_7hrs	11/707	0.03	-1.78	6.26	GBP6;IL13RA1;RASSF4;PLEK;RAB32; TLR6;GBP3;ZYX;IFNGR2;GM885;IRF5
macrophage_peri_LPS_thio_0hrs	6/353	0.06	-1.55	4.28	NPC1;5430435G22RIK;MPEG1; AHNAK;PSTPIP1;SNX27
dendritic_plasmacytoid_B220+	4/214	0.06	-1.43	3.95	PLD4;RCBTB2;CTSH;DTX2

macrophage_peri_LPS_thio_1hrs	9/598	0.24	-1.83	2.58	NPC1;TNFAIP3;AHNAK;SNX27;5430435G22RIK;MPEG1;PLEK;MCL1;IFNGR2
dendritic_cells_lymphoid_CD8a+	4/142	0.12	-1.07	2.26	SLC12A6;IDH2;HPS3;CCDC88A
stem_cells__HSC	2/186	0.19	-1.28	2.15	WDR67;FOS

Supplementary Table 3. Peak set *de novo* motif analysis

PU.1 Group 1

Motif Family	P-value	% of Targets	% of Background	Best Match/Details
ETS	1e-808	70.93%	9.86%	PB0058.1_Sfpi1_1/Jaspar 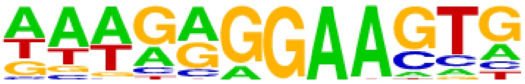
CEBP	1e-154	35.99%	11.51%	MF0006.1_bZIP_cEBP-like_subclass/Jaspar 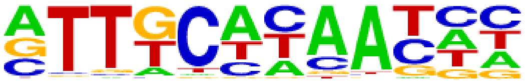
ISRE	1e-61	16.15%	5.24%	ISRE(IRF)/ThioMac-LPS-exp/HOMER 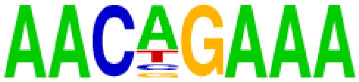
RUNX	1e-38	20.65%	10.00%	RUNX2(Runt)/PCa-RUNX2-ChIP-Seq(GSE33889)/Homer 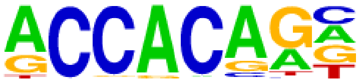
ISRE	1e-35	15.46%	6.75%	IRF4(IRF)/GM12878-IRF4-ChIP-Seq/Homer 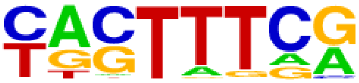
MyoG	1e-22	10.78%	4.89%	MyoG(HLH)/C2C12-MyoG-ChIP-Seq(GSE36024)/Homer 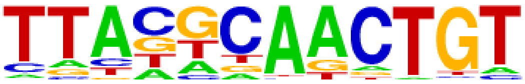
AP1	1e-19	11.53%	5.71%	Jun-AP1(bZIP)/K562-cJun-ChIP- 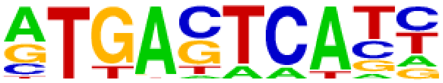

PU.1 Group II

Motif Family	P-value	% of Targets	% of Background	Best Match/Details
ETS	1e-2107	64.70%	8.76%	PB0058.1_Sfpi1_1/Jaspar 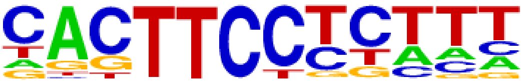
CEBP	1e-242	26.44%	9.95%	MF0006.1_bZIP_cEBP-like_subclass/Jaspar 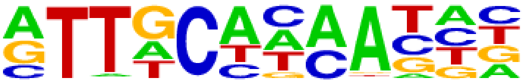
PRDM1	1e-214	19.01%	6.08%	PRDM1/BMI1(Zf)/Hela-PRDM1-ChIP-Seq(GSE31477)/Homer 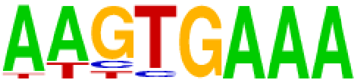
RUNX	1e-139	15.70%	5.80%	RUNX(Runt)/HPC7-Runx1-ChIP-Seq/Homer 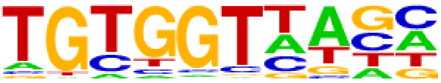
ETS-RUNX	1e-86	5.54%	1.31%	ETS:RUNX/Jurkat-RUNX1-ChIP-Seq/Homer 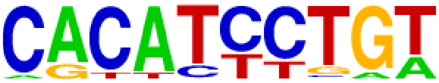
AP-1	1e-38	5.80%	2.47%	AP-1(bZIP)/ThioMac-PU.1-ChIP-Seq/Homer 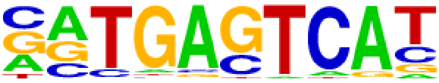

PU.1 Group III

Motif Family	P-value	% of Targets	% of Background	Best Match/Details
ETS	1e-718	54.58%	10.37%	ETS1(ETS)/Jurkat-ETS1-ChIP-Seq/Homer 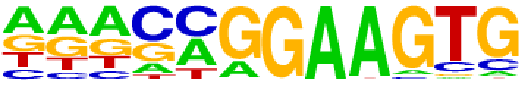
CEBP	1e-311	26.11%	4.73%	MF0006.1_bZIP_cEBP-like_subclass/Jaspar 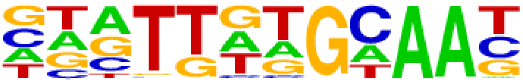
RUNX	1e-128	27.38%	10.80%	RUNX2(Runt)/PCa-RUNX2-ChIP-Seq(GSE33889)/Homer 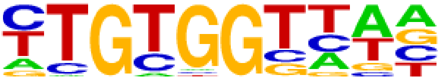
Pol	1e-53	20.21%	10.24%	POL003.1_GC-box/Jaspar 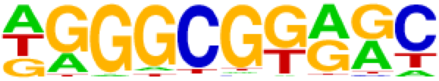
AP-1	1e-44	7.90%	2.60%	AP-1(bZIP)/ThioMac-PU.1-ChIP-Seq/Homer 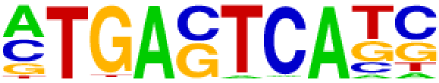
ZF1	1e-33	8.55%	3.55%	BORIS(Zf)/K562-CTCFL-ChIP-Seq/Homer 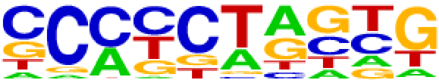
NRF	1e-25	7.28%	3.20%	NRF1(NRF)/MCF7-NRF1-ChIP-Seq/Homer 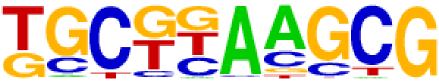
CRE	1e-23	16.91%	10.50%	CRE(bZIP)/Promoter/Homer 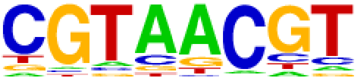
ISRE	1e-20	6.12%	2.73%	ISRE(IRF)/ThioMac-LPS-exp/HOMER 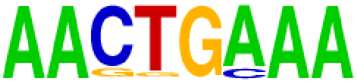
